# Outpatient satisfaction assessed using a nationally standardized questionnaire: a survey of five health care facilities in Vietnam

**DOI:** 10.3389/frhs.2026.1844842

**Published:** 2026-08-06

**Authors:** Kieu-Nga Thi Dang, Nguyet Anh Quang, Mi Ha Nguyen, Nhan Bich Huynh, Nhan Thanh Nguyen, Thanh-Truc Tran Pham, Trung Hoang Mai, Nga Thi-Quynh Nguyen, Hai-Yen Thi Nguyen

**Affiliations:** 1 School of Pharmacy, University of Medicine and Pharmacy at Ho Chi Minh City, Ho Chi Minh City, Vietnam; 2 Tuy Phong Regional Health Center, Binh Thuan, Vietnam; 3 Nhan Hau Phu Cuong Clinic, An Giang, Vietnam; 4 Vinh Cuu Regional Health Center, Dong Nai, Vietnam; 5 Saigon ITO Phu Nhuan Hospital, Ho Chi Minh City, Vietnam; 6 Minh Anh International Hospital, Ho Chi Minh City, Vietnam

**Keywords:** CFA, healthcare quality, outpatient, patient satisfaction, SEM, Vietnam

## Abstract

**Background:**

Patient satisfaction surveys are pivotal for evaluating healthcare quality. This study aims to validate the Vietnamese national standard outpatient satisfaction tool, assess outpatient satisfaction level, and identify key factors associated with satisfaction across multiple healthcare facilities.

**Methods:**

A multi-center cross-sectional survey was conducted between October 2024 and September 2025 among 2,500 outpatients at five healthcare facilities. The nationally standardized questionnaire comprised 31 items across five dimensions: Accessibility, Procedures, Facilities, Personnel and Outcomes. Data were analyzed using descriptive statistics, ANOVA, and Structural Equation Modeling (SEM). Harman's single-factor test was applied to assess common method variance.

**Results:**

A total of 2,500 valid responses were analyzed, with 54.0% of participants being female. The mean satisfaction score was 3.87 ± 0.49. The structural model showed a good fit and identified Personnel (*β* = 0.43) and Accessibility (*β* = 0.30) as the most significant factors associated with outpatient satisfaction (*p* < 0.001). Outcomes also showed a significant positive association (*β* = 0.14, *p* < 0.01), whereas Procedures and Facilities did not yield statistically significant impacts (*p* > 0.05). Harman's test confirmed no significant common method bias (37.43% variance). Higher satisfaction levels were significantly associated with health insurance holders, rural residents, and repeat visitors (*p* < 0.05).

**Conclusion:**

The 2024 national standardized questionnaire is a valid and reliable tool for assessing healthcare quality in Vietnam. To improve outpatient experiences, healthcare providers should prioritize enhancing staff communication skills and service accessibility. These findings support the institutionalization of patient-centered metrics to drive sustainable quality management in resource-constrained environments.

## 1 Introduction

Patient satisfaction is a key indicator of healthcare quality and system performance. In a patient-centered model, it reflects service responsiveness and efficiency, while being directly linked to better treatment adherence and clinical outcomes, forming the foundation of sustainable healthcare management (1). Standardized frameworks like HCAHPS in the United States and the Picker Patient Experience Questionnaire in Europe have enabled global benchmarking and data-driven quality improvements (2). Similarly, many developing Asian nations are integrating patient feedback into national health strategies to align rapid infrastructure expansion with perceived quality of care (3–5).

In Vietnam, the Ministry of Health has prioritized elevating patient satisfaction through decisive regulatory measures. A significant milestone was Decision No. 4276/QD-BYT (2015), which established the National Action Program to enhance healthcare quality management through 2025 (6). This policy emphasized using satisfaction indices and public perceptions to propose evidence-based solutions. Recently, the framework was updated via Decision No. 56/QD-BYT on January 8, 2024 (7). This mandate introduced standardized survey templates for assessing the satisfaction of both patients and healthcare providers, officially superseding models from Decision No. 4448/QD-BYT (2013) and Decision No. 3869/QD-BYT (2019) (8). This transition reflects Vietnam's commitment to refining measurement methodologies within a rapidly evolving medical landscape. Despite growing literature on patient satisfaction, most studies are limited by small sample sizes or focus on single institutions and specific patient groups (9–12). There is a notable lack of evidence-based research evaluating diverse healthcare models simultaneously—from private international hospitals in urban centers to public regional health centers and provincial clinics. Although the Ministry of Health recently issued an updated national standardized patient satisfaction questionnaire in 2024 (7), there is a lack of empirical research applying this latest instrument across diverse geographical and administrative contexts. Furthermore, few studies have employed robust statistical methods like Structural Equation Modeling (SEM) to validate this 2024 version. Understanding satisfaction drivers through this updated lens is essential for mitigating systemic bottlenecks that vary between high-tech urban facilities and resource-constrained local centers (11, 13).

This study aims to address these gaps by analyzing a sample of 2,500 outpatients to identify key satisfaction determinants. Using a nationally standardized questionnaire, the research examines how demographic and service-related factors contribute to the perceived quality of care, offering actionable recommendations for healthcare improvement in Vietnam.

## 2 Method

### 2.1 Study design and setting

This study employed a multi-center, cross-sectional survey design to assess outpatient satisfaction. The research was conducted across five diverse healthcare facilities in Vietnam, providing a representative mix of the national health system. These included two private hospitals in Ho Chi Minh City (Minh Anh International Hospital and Saigon ITO Phu Nhuan Hospital), two public health centers (Tuy Phong Regional Health Center in Lam Dong Province and Vinh Cuu Regional Health Center in Dong Nai Province), and one private clinic (Nhan Hau Phu Cuong Clinic in An Giang Province). Collectively, these facilities operate with a total capacity of approximately 850 beds and serve nearly 1.2 million outpatient visits annually. The outpatient departments across these sites follow a standardized clinical workflow consisting of four primary stages: (i) Registration and vital signs assessment; (ii) Clinical consultation with specialists; (iii) Paraclinical investigations; and (iv) Prescription issuance, payment, and medication dispensing. The study was reported following the Strengthening the Reporting of Observational Studies in Epidemiology (STROBE) guidelines (14).

### 2.2 Participants and study procedures

The patient satisfaction monitoring system has been operational in these facilities since 2019 and was updated to align with the new national standardized tool in early 2024. Data were collected between October 2024 and September 2025.

Eligible participants included all outpatients who had completed the clinical examination process and were either waiting for payment or medication. Convenience sampling was applied to recruit participants. Following an explanation of the study's objectives, participants provided written informed consent before completing the survey via a paper-based questionnaire or a digital interface (tablet/smartphone) accessed via a QR code. Participation was entirely voluntary, and anonymity was strictly maintained to minimize social desirability bias (15). To ensure data completeness, all paper-based questionnaires were reviewed by trained survey personnel immediately after completion. Whenever feasible, respondents were invited to complete unintentionally omitted items before leaving the survey site. For electronic questionnaires, mandatory-response settings prevented submission of incomplete questionnaires.

### 2.3 Sample size determination

The sample size was determined following the national standards for healthcare quality appraisal as mandated in Decision No. 56/QD-BYT (2024) by the Vietnam Ministry of Health (7). For each facility, the minimum required sample (n) was calculated using the formula for a single proportion in cross-sectional studies: $n=Z_{\left(1-\frac{\alpha }{2}\right)}^{2}.\frac{\;p(1-p)}{d^{2}}$, where $Z_{\left(1-\frac{\alpha }{2}\right)}^{1}=1.96$ (95% confidence level), *p* = 0.85 (expected satisfaction rate), and d = 0.05 (absolute precision).

Following the national guideline, a finite population correction was applied based on the daily outpatient volume of each facility: *n*_adj_ = *n*/[1 + (*n*−1)/*N*], where *N* denotes the number of outpatient visits per day at each participating healthcare facility, as defined in Decision No. 56/QD-BYT (2024). The calculated sample size represented the minimum number of participants required for each facility. However, participant recruitment was not terminated after the minimum required sample size had been reached. Instead, data collection continued throughout the predefined study period (October 2024 to September 2025), and all eligible completed questionnaires were included in the analysis. Consequently, the final analytical sample comprised 2,500 completed questionnaires, substantially exceeding the minimum required sample size. The average number of outpatient visits per day (*N*), the corresponding minimum required sample size for each participating healthcare facility, and the actual number of recruited participants are presented in Supplementary Table S1.

### 2.4 Measuring tool

The outpatient satisfaction questionnaire was promulgated by the Vietnam Ministry of Health as the standardized instrument for national healthcare quality appraisal under Decision No. 56/QD-BYT (2024) (7). The questionnaire consists of three primary components (described in Supplementary Table S2). First, the general and socio-demographic information captures the context of the survey and patient characteristics, incorporating items such as date of completion, survey administrator (interviewer/person completing the questionnaire), respondent, gender, age, distance to the facility, health insurance status, residential area, economic status, and visit status. In this study, these socio-demographic and visit-related characteristics serve as the primary factors to identify potential associations with patient experiences. Second, the satisfaction survey comprises five dimensions with 31 items: “Accessibility”, “Transparency of information and procedures” (Procedures), “Facilities and equipment” (Facilities), “Provider attitudes and competence” (Personnel), and “Service outcomes” (Outcomes). All satisfaction items were rated on a five-point Likert scale ranging from 1 (very dissatisfied) to 5 (very satisfied). These five dimensions function as the core dependent variables. The primary outcome (Patient Satisfaction) is operationalized as a composite score, calculated as the arithmetic mean of these five dimensions. Finally, it includes two general satisfaction items, namely, “Patient's overall evaluation of this hospital” (Overall evaluation), “Return or recommendation of this hospital to others” (Recommendation). These items are utilized as indicator variables to validate the convergence between the calculated composite satisfaction score and the patient's subjective global perception. Given that this is a newly updated national instrument, its psychometric properties, including structural validity and internal consistency, were further validated in this study using SEM and Cronbach's alpha.

### 2.5 Statistical analysis

#### 2.5.1 Data preparation

Data from the five facilities were merged into a centralized database. Following the quality-control procedures implemented during data collection, all 2,500 collected questionnaires were complete and contained no missing responses; therefore, the entire sample (*N* = 2,500) was retained for analysis. For each respondent, a total satisfaction score was obtained by averaging the scores of all satisfaction items.

#### 2.5.2 Data analysis

Statistical analyses were performed using IBM SPSS 27.0 and AMOS 24.0. Descriptive statistics, including frequencies, percentages, means and standard deviations (SD), were used to summarize socio-demographic characteristics and outpatient satisfaction levels across five dimensions.

Prior to group comparisons, normality tests were conducted to determine the appropriate statistical approach. For normally distributed data, one-way ANOVA was employed to identify significant differences in satisfaction scores across demographic variables. In cases where the assumption of homogeneity of variances was violated, the Welch ANOVA test was utilized, while the Kruskal–Wallis test served as a non-parametric alternative for data that did not meet the normality criteria. In these group-comparison analyses, socio-demographic factors (e.g., gender, age, insurance status) were treated as the independent variables, while the five satisfaction dimensions and the composite satisfaction score were the dependent variables. When the group-comparison was statistically significant, *post hoc* pairwise comparisons were performed using Tukey's honestly significant difference (HSD) test following one-way ANOVA, Games–Howell following Welch ANOVA, and Bonferroni-adjusted pairwise comparisons following the Kruskal–Wallis test.

The study further adopted a two-step SEM approach. In the first step, Confirmatory Factor Analysis (CFA) was conducted to validate the measurement model using the 31 observed satisfaction items representing the five latent constructs. Given the large sample size (*N* = 2,500) and the approximately normal distribution of the observed variables, the Maximum Likelihood estimation method was applied. As a preliminary assessment of the inter-item correlation structure, the Kaiser–Meyer–Olkin (KMO) measure and Bartlett's test of sphericity were calculated prior to confirmatory factor analysis. The internal consistency of the scales was assessed using Cronbach's alpha. The model fit was primarily evaluated using the commonly recommended fit indices, including the Minimum Discrepancy per Degree of Freedom (CMIN/df), Comparative Fit Index (CFI), Tucker–Lewis Index (TLI), and Root Mean Square Error of Approximation (RMSEA). Additional fit indices were also reported for completeness. The recommended thresholds were CMIN/df ≤ 3.0, CFI ≥ 0.90, TLI ≥ 0.90, and RMSEA ≤ 0.06 (16). These criteria ensure that the structural model appropriately represents the observed relationships within the dataset. Model modifications were performed sequentially based on modification indices. Item-level method effects, implemented by specifying correlated error terms, were incorporated only when supported by both modification indices and clear theoretical justification based on conceptual overlap between item contents. Standardized factor loadings were reported to verify the relationships between latent constructs and their respective items. In the second step, the structural model was tested to identify the primary factors associated with Patient satisfaction through standardized regression weights, while the two global satisfaction items (Overall evaluation and Recommendation) were incorporated to examine their relationships with the latent construct. To minimize potential bias and address confounding, facility type (public vs. private) was incorporated into the analysis as a confounding variable to ensure that observed variations in satisfaction were not disproportionately attributed to institutional differences. Finally, Harman's single-factor test was applied to assess common method variance (15), and all statistical tests were two-tailed with a significance level of *p* < 0.05.

## 3 Result

### 3.1 Socio-demographic characteristics of patients

A total of 2,500 outpatients participated in the study, with the socio-demographic profile summarized in Table 1. The study population showed a broad age distribution, with the largest proportion being middle-aged adults (40–59 years, 38.0%), followed by young adults (18–39 years, 29.2%) and older adults (60–79 years, 24.8%). Most participants were female (54.0%), and utilized health insurance (68.8%). The majority were repeat visitors (80.0%) living within 5 km of the facility (53.9%). Regarding the healthcare facility, the sample was distributed across five facilities, encompassing both the public sector (40.0%) and the private sector (60.0%). Data collection was predominantly self-administered (92.7%) and reached its peak in the third quarter (33.2%).

**Table 1:** Socio-demographic characteristics of the participants.

Socio-demographic	Count	Percent
Quarter (data collected)		
The first quarter	625	25.0%
The second quarter	625	25.0%
The third quarter	829	33.2%
The fourth quarter	421	16.8%
Survey Administrator		
Patient self-administered	2,317	92.7%
Healthcare staff	183	7.3%
Respondent		
Patient	2,053	82.1%
Caregiver	447	17.9%
Gender		
Female	1,350	54.0%
Male	1,150	46.0%
Insurance status for current visit		
No	779	31.2%
Yes	1,721	68.8%
Residence		
Urban	1,363	54.5%
Rural	1,031	41.2%
Remote/Disadvantaged areas	106	4.2%
Economic status		
Poor	45	1.8%
Near poor	166	6.6%
Others	2,289	91.6%
Age (Group)		
<18	23	0.9%
18–39	730	29.2%
40–59	951	38.0%
60–79	735	29.4%
≥80	61	2.4%
Distance (Group)		
<2 km	443	17.7%
2–<5 km	906	36.2%
5–<10 km	631	25.2%
≥10 km	520	20.8%
Visit status		
First-time visit	500	20.0%
Repeat visit	2,000	80.0%
Healthcare Facility		
***Private Sector***	***1,500***	***60.0%***
Minh Anh International Hospital	500	20.0%
Saigon ITO Phu Nhuan Hospital	500	20.0%
Nhan Hau Phu Cuong Clinic	500	20.0%
***Public Sector***	***1,000***	***40.0%***
Tuy Phong Regional Health Center	500	20.0%
Vinh Cuu Regional Health Center	500	20.0%

Bold values indicate subgroup totals for private-sector and public-sector healthcare facilities.

### 3.2 Structural equation modelling analysis

Valid responses (*n* = 2,500) covered the full scale range (1–5) and were normally distributed, with a mean (SD) of 3.87 (0.49).

#### 3.2.1 Validity and reliability

As a preliminary assessment, the KMO value was 0.964 and Bartlett's test of sphericity was statistically significant (*p* < 0.001), indicating sufficient inter-item correlations to proceed with confirmatory factor analysis. The scale exhibited high internal consistency (Cronbach's alpha = 0.944). Additionally, Harman's single-factor test showed that the first factor accounted for 37.43% of the total variance, well below the 50% threshold, indicating that common method bias was not a significant concern.

#### 3.2.2 Evaluation of measurement and structural models

##### 3.2.2.1 Measurement model fit

**Figure 1:**
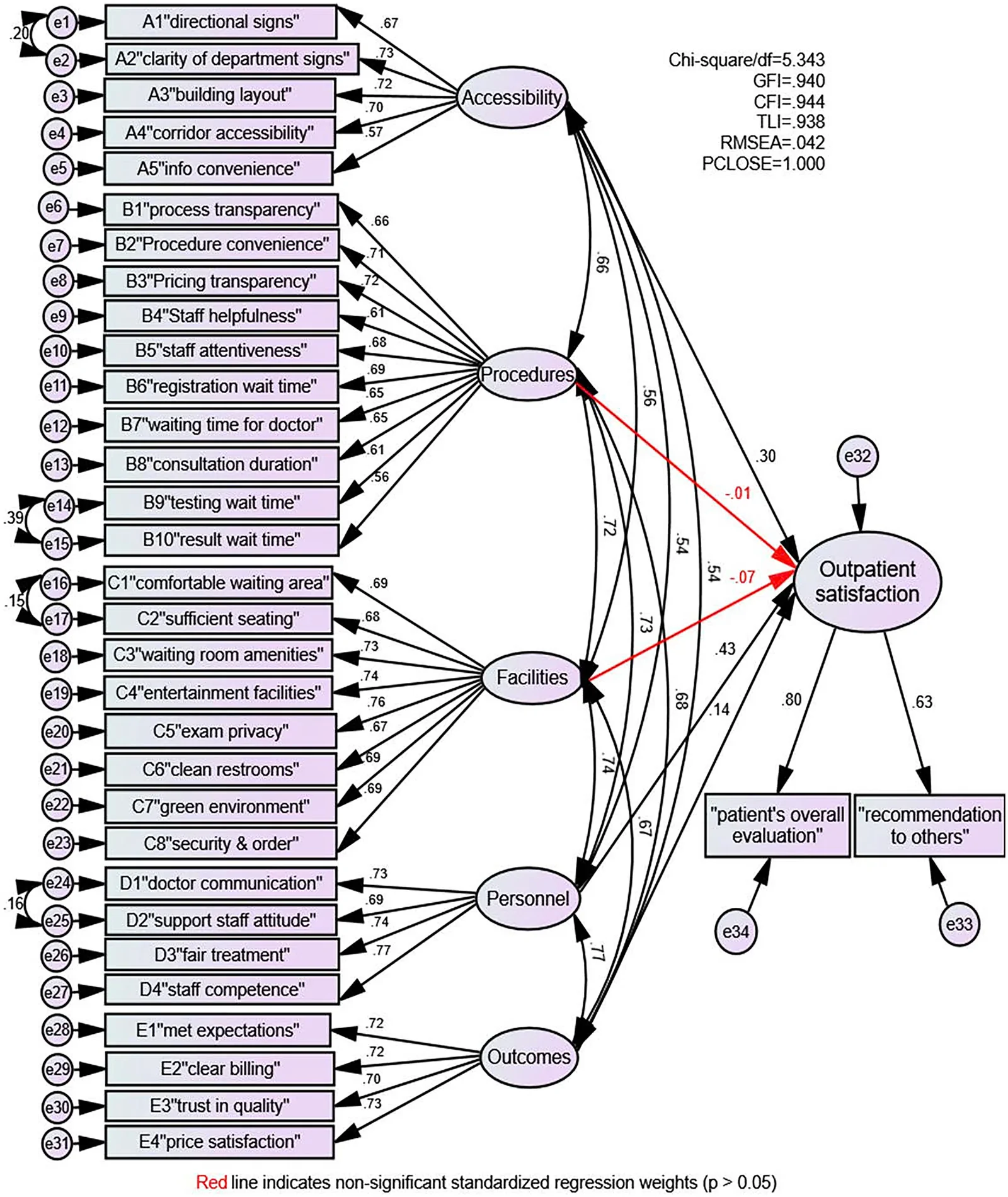
Structural equation model and standard regression weights of outpatient satisfaction.

The measurement model demonstrated an acceptable fit to the data, with the primary fit indices indicating good model fit (CMIN/df = 5.343, CFI = 0.944, TLI = 0.938, and RMSEA = 0.042). Additional model fit statistics are presented in Table 2. All observed variables showed statistical significance (*p* < 0.001). As illustrated in Figure 1, the five latent factors were inter-correlated at moderate to strong levels, with coefficients ranging from 0.54 to 0.77. All correlations remained below the 0.85 threshold, confirming the discriminant validity of the constructs. Four item-level method effects were modeled by specifying correlated error terms between item pairs with closely related content: A1–A2 (hospital signage and wayfinding), B9–B10 (waiting-time experiences during the diagnostic process), C1–C2 (waiting-area environment), and D1–D2 (communication with healthcare personnel). These item-level method effects were considered conceptually justified because each item pair addressed highly related aspects of the same latent construct. No additional item-level method effects were specified to minimize the risk of model overfitting.

**Table 2:** Model fit indices.

Indices	Value	Indices	Value
CMIN/DF	5.343	GFI	0.940
CFI	0.944	AGFI	0.930
TLI	0.938	NFI	0.932
RMSEA	0.042	RFI	0.925
PCLOSE	1.000	IFI	0.944
RMR	0.038	PGFI	0.799

##### 3.2.2.2 Structural model results

The structural model (Figure 1) identified “Personnel” (*β* = 0.43) and “Accessibility” (*β* = 0.30) as the most significant factors associated with outpatient satisfaction (*p* < 0.001), followed by “Outcomes” (*β* = 0.138, *p* < 0.01). Conversely, the associations for “Procedures” (*β* = −0.01) and “Facilities” (*β* = −0.07) were not statistically significant to outpatient satisfaction (*p* > 0.05).

### 3.3 Satisfaction level of outpatients

**Figure 2:**
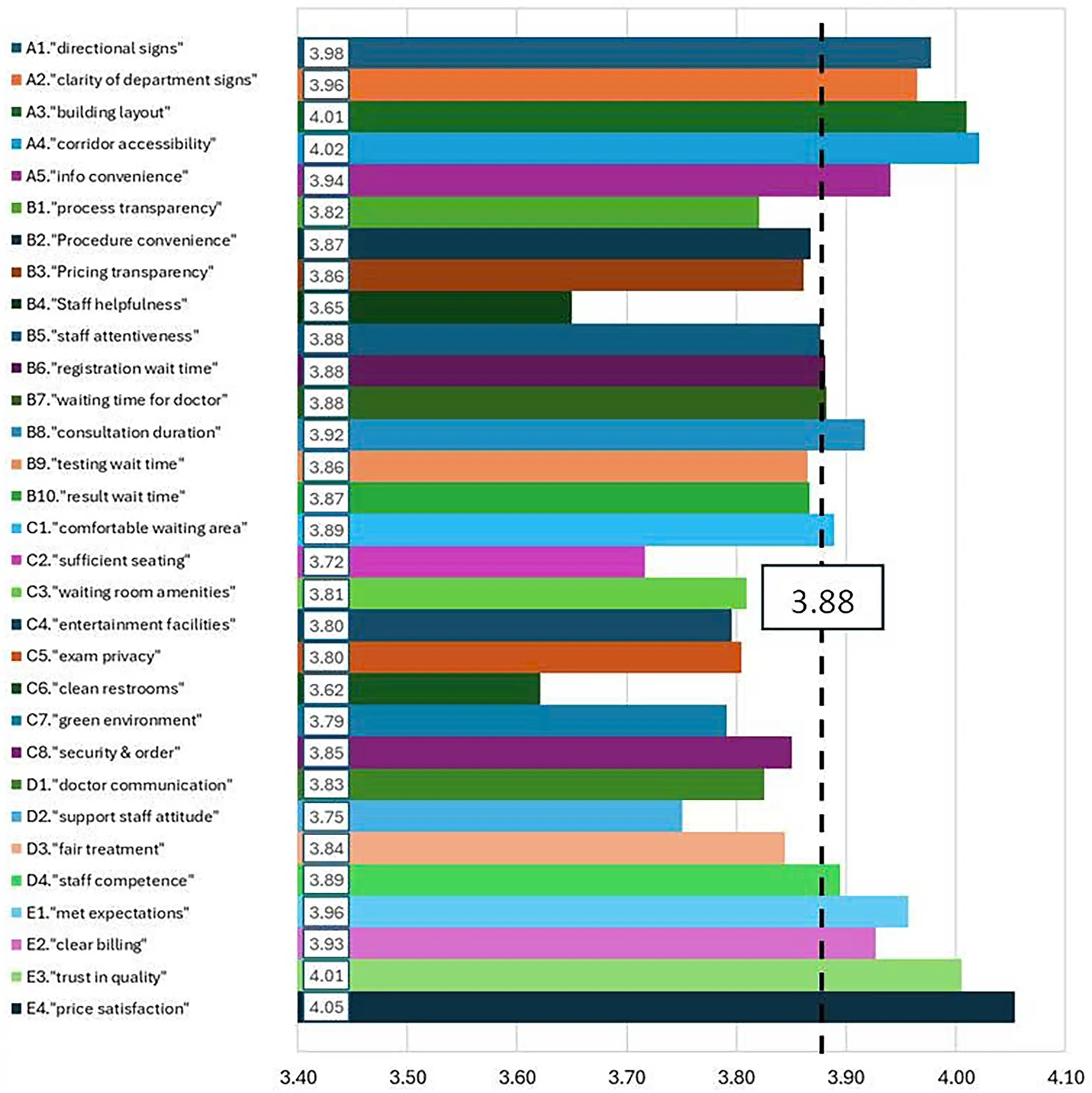
Means of satisfaction items.

The results of the “patient's overall evaluation of this hospital” (overall evaluation) and specific satisfaction dimensions are summarized in Supplementary Table S3. The mean (SD) score for the overall evaluation was 77.59% (15.34%). Notably, 43.32% of the patients rated their satisfaction above 80% (very high satisfaction). A similar key indicator, “return or recommendation of this hospital to others” (recommendation level), yielded a mean score of 4.30 (0.85) (details are described in Supplementary Table S3). The mean satisfaction scores for individual dimensions and items are illustrated in Figure 2. When the “Overall satisfaction” (mean = 3.88) was established as the reference value, the highest-performing items were concentrated in Accessibility (Domain A) and Outcomes (Domain E). Specifically, “price satisfaction”, “institutional trust”, and “navigational infrastructure” consistently surpassed this threshold. High professional scores were also observed for “consultation duration” and “staff competence”. “Price satisfaction” from Outcomes recorded the highest mean of 4.05, whereas “clean restrooms” from Facilities recorded the lowest mean of 3.62 (details of these satisfaction questions are listed in Supplementary Table S3).

### 3.4 Association between socio-demographics factors and general satisfaction levels

The three key outcome measures—Overall evaluation, Recommendation level and the Total score—were compared across patient socio-demographic characteristics (Table 3). Statistical analysis revealed that satisfaction levels varied significantly based on several contextual factors (*p* < 0.05). Specifically, higher satisfaction and recommendation scores were consistently associated with the third quarter, self-administered surveys, health insurance holders, rural residents, and repeat visitors. While overall satisfaction and total scores exhibited minor quarterly fluctuations—likely reflecting seasonal variations in service delivery—the Recommendation level remained consistently high and stable across all periods. Furthermore, a substantial variation in satisfaction was observed across different healthcare facilities (*p* < 0.001), with public health centers generally reporting higher mean scores in overall evaluation compared to private hospitals and clinics.

**Table 3:** General satisfaction indicators grouped by demographics.

Values	Overall evaluation		Recommendation level			Total score	
	Mean ± SD	*p*-value	Median (IQR)	Mean Rank	*p*-value	Mean ± SD	*p*-value
Quarter (data collected)							
The first quarter	3.83 ± 0.77^ab^	<0.001^2***^	4.00 (1.00)^ab^	1,245.63	0.015^3*^	3.74 ± 0.47	<0.001^2***^
The second quarter	3.93 ± 0.80^ac^		4.00 (1.00)^ab^	1,261.26		3.83 ± 0.49	
The third quarter	3.94 ± 0.69^c^		4.00 (1.00)^a^	1,289.43		3.87 ± 0.43	
The fourth quarter	3.75 ± 0.83^b^		4.00 (1.00)^b^	1,165.11		4.11 ± 0.57	
Survey Administrator							
Patient self-administered	3.91 ± 0.77	<0.001^2***^	4.00 (1.00)	1,295.35	<0.001^3***^	3.87 ± 0.50	0.581^2^
Healthcare staff	3.52 ± 0.59		4.00 (2.00)	682.67		3.85 ± 0.42	
Respondent							
Patient	3.90 ± 0.76	0.005^1**^	5.00 (1.00)	1,313.40	<0.001^3***^	3.85 ± 0.49	<0.001^1***^
Caregiver	3.79 ± 0.80		4.00 (1.00)	961.60		3.96 ± 0.50	
Gender							
Female	3.87 ± 0.79	0.642^1^	5.00 (1.00)	1,243.10	0.539^3^	3.86 ± 0.51	0.649^1^
Male	3.89 ± 0.74		4.00 (1.00)	1,259.18		3.87 ± 0.48	
Insurance status for current visit							
No	3.75 ± 0.83	<0.001^2***^	4.00 (1.00)	919.51	<0.001^3***^	3.94 ± 0.50	<0.001^2***^
Yes	3.94 ± 0.73		5.00 (1.00)	1,400.32		3.84 ± 0.48	
Residence							
Urban	3.85 ± 0.80^a^	<0.001^2***^	4.00 (1.00)^a^	1,208.72	0.001^3***^	3.87 ± 0.52	0.144^2^
Rural	3.94 ± 0.71^b^		5.00 (1.00)^b^	1,308.09		3.87 ± 0.45	
Remote/Disadvantaged areas	3.66 ± 0.82^a^		4.00 (1.00)^ab^	1,227.64		3.77 ± 0.55	
Economic status							
Poor	3.96 ± 0.85	0.629^1^	4.00 (1.00)^ab^	1,274.03	0.003^3**^	4.05 ± 0.63^ab^	<0.001^2***^
Near poor	3.92 ± 0.83		4.00 (1.00)^a^	1,082.55		4.06 ± 0.55^a^	
Others	3.88 ± 0.76		4.00 (1.00)^b^	1,262.22		3.85 ± 0.48^b^	
Age (Group)							
<18	3.96 ± 0.64	0.691^2^	4.00 (1.00)	1,136.89	0.286^3^	4.03 ± 0.39^ab^	<0.001^2***^
18–39	3.87 ± 0.80		4.00 (1.00)	1,244.90		3.83 ± 0.50^a^	
40–59	3.86 ± 0.75		4.00 (1.00)	1,275.42		3.85 ± 0.49^a^	
60–79	3.91 ± 0.75		4.00 (1.00)	1,238.58		3.93 ± 0.47^b^	
≥80	3.83 ± 0.91		4.00 (1.00)	1,115.42		3.97 ± 0.60^ab^	
Distance (Group)							
<2 km	3.85 ± 0.82	0.356^2^	5.00 (1.00)^a^	1,551.32	<0.001^3***^	3.69 ± 0.46^a^	<0.001^2***^
2–<5 km	3.87 ± 0.83		5.00 (1.00)^b^	1,326.35		3.81 ± 0.49^b^	
5–<10 km	3.87 ± 0.74		4.00 (1.00)^c^	996.26		3.98 ± 0.47^c^	
≥10 km	3.93 ± 0.64		4.00 (1.00)^d^	1,170.58		3.99 ± 0.49^c^	
Visit status							
First-time visit	3.67 ± 0.86	<0.001^2***^	4.00 (1.00)	1,055.48	<0.001^3***^	3.78 ± 0.56	<0.001^2***^
Repeat visit	3.93 ± 0.73		5.00 (1.00)	1,299.25		3.89 ± 0.47	
Healthcare Facility							
Minh Anh International Hospital	3.63 ± 0.84^a^	<0.001^2***^	4.00 (1.00)^a^	808.23	<0.001^3***^	3.88 ± 0.48^a^	<0.001^2***^
Saigon ITO Phu Nhuan Hospital	3.91 ± 0.73^b^		4.00 (0.00)^b^	947.66		4.18 ± 0.56^b^	
Nhan Hau Phu Cuong Clinic	3.37 ± 0.89^c^		5.00 (1.00)^c^	1,448.29		3.50 ± 0.46^c^	
Tuy Phong Regional Health Center	4.33 ± 0.41^d^		5.00 (0.00)^d^	1,787.12		3.68 ± 0.23^d^	
Vinh Cuu Regional Health Center	4.15 ± 0.39^e^		4.00 (1.00)^e^	1,261.20		4.09 ± 0.31^e^	

1 One-way ANOVA.

2 Welch ANOVA.

3 Kruskal–Wallis test.

* *p* < 0.05.

** *p* < 0.01.

*** *p* < 0.001.

Post hoc comparisons were performed using Games–Howell following Welch ANOVA, and Bonferroni-adjusted pairwise comparisons following the Kruskal–Wallis test. Different superscript letters indicate statistically significant pairwise differences (*p* < 0.05); groups sharing at least one letter are not significantly different.

Superscript letters (a–e) indicate the results of post hoc pairwise comparisons. Groups sharing at least one superscript letter are not significantly different, whereas groups with no superscript letter in common are significantly different (*p* < 0.05).

Regarding personal characteristics, while gender showed no significant impact, both age and economic status were significantly associated with the Recommendation level (*p* < 0.05) and the Total score (*p* < 0.001), Notably, the highest satisfaction levels were recorded among the elderly (aged ≥ 80) and patients in the Near-poor group, suggesting specific demographic trends in service perception.

**Figure 3:**
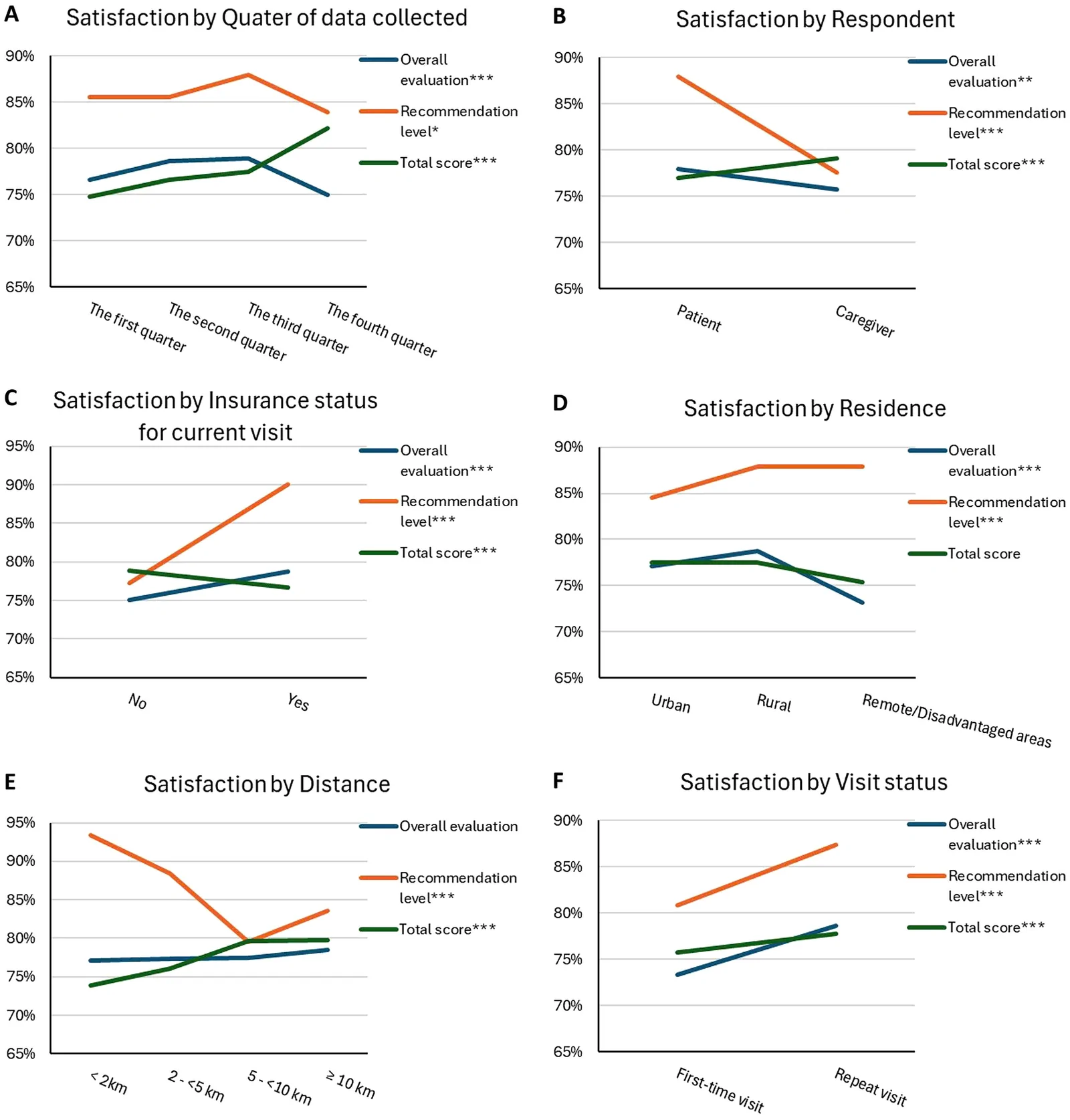
Corresponding percentage of the means of general satisfaction indicators. **(A)** Quarter (data collected); **(B)** respondent; **(C)** insurance status for current visit; **(D)** residence; **(E)** distance; **(F)** visit status.

These trends are further illustrated in Figure 3, which highlights a consistent pattern: the Recommendation level consistently surpassed both the Overall evaluation and Total score across nearly all demographic categories. More detailed comparisons across all socio-demographic factors are provided in Supplementary Figure S1.

### 3.5 Subgroup analysis of key satisfaction-related factors

**Figure 4:**
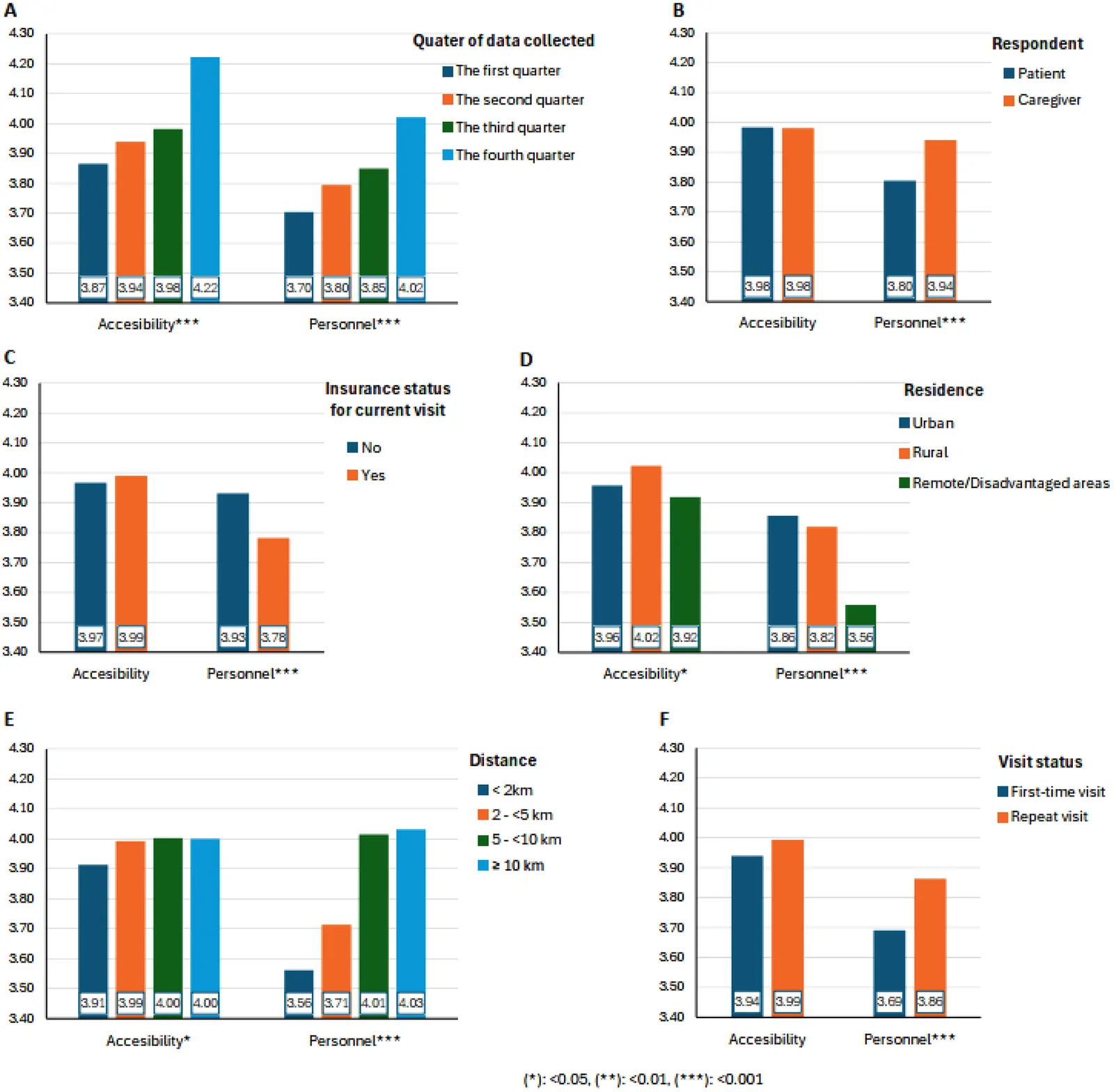
Comparison of the sum scores of the two primary factors. **(A)** Quarter (data collected); **(B)** respondent; **(C)** insurance status for current visit **(D)** residence; **(E)** distance; **(F)** visit status.

Although Outcomes also showed a significant positive association with satisfaction, the subgroup analysis focused on Personnel and Accessibility as they demonstrated the strongest correlations within the structural model. Figure 4 illustrates the variations of these two key factors across six primary characteristics, while the complete breakdown of all analyzed variables is detailed in Supplementary Figure S2.

Higher mean scores for Accessibility were consistently observed among rural residents and repeat visitors. Notably, while Personnel scores were significantly lower among patients with health insurance compared to those without, they showed a positive trend with geographical distance, reaching the highest levels among patients traveling ≥ 10 km (*p* < 0.001).

Conversely, the lowest scores for Personnel were reported by patients residing within 2 km of the facility and those from remote or disadvantaged areas. While temporal factors (Quarter) significantly associated with both domains (*p* < 0.001), insurance status showed a significant association only with Personnel (*p* < 0.001). These findings suggest that while physical accessibility is perceived relatively consistently across groups, the perception of personnel interaction is more sensitive to the patient's financial status and their residential background.

## 4 Discussion

This study represents a comprehensive evaluation of outpatient satisfaction across five diverse healthcare facilities in Vietnam, utilizing a nationally standardized questionnaire. Through rigorous analysis of validity and reliability, we confirmed that the survey instrument consistently adhered to the established framework. The study successfully identified the primary influencing factors: personnel, accessibility, outcomes, residence, insurance status, and visit status. These findings are supported by a substantial dataset, providing a robust baseline for quality improvement in the Vietnamese healthcare context.

### 4.1 Key model implications and factors related to patient satisfaction

The utilization of a standardized questionnaire has resulted in a sample exhibiting favorable characteristics of reliability and validity. While the CMIN/df (5.343) exceeded the traditional threshold of 3.0, this is frequently observed in large-scale studies (*N* = 2,500), as the Chi-square statistic is highly sensitive to large sample sizes (17). In such cases, the model fit is more accurately reflected by indices less dependent on sample size, such as GFI (0.940), CFI (0.944), TLI (0.938), all of which surpassed the recommended 0.90 cutoff (16). Furthermore, the PGFI (0.799) confirms that the model's complexity is well-balanced with its fit, suggesting that the selected factors efficiently explain the variance in satisfaction without over-complication.

Building on this robust model, the structural analysis identifies Personnel, Accessibility, and Outcomes as the factors most significantly associated with patient satisfaction, aligning with Batbaatar et al. (2017), whose global review confirmed interpersonal care and accessibility as universal predictors (18). Specifically, the primary contribution of Personnel (*β* = 0.43, *p* < 0.001) echoes Nguyen et al. (2022) regarding staff expertise in Vietnam, while the association of Accessibility (*β* = 0.30, *p* < 0.001) parallels Hoang et al. (2023), highlighting administrative efficiency as a key differentiator in multi-center settings (10, 12). Furthermore, while Outcomes showed a significant positive association (*β* = 0.14, *p* < 0.01), its lower coefficient suggests that in the outpatient context, the human interaction and ease of access often outweigh the perceived service result in shaping immediate satisfaction. In contrast, the non-significant associations of Facilities and Procedures (*p* > 0.05) suggests these act as hygiene factors. According to Batbaatar et al. (2017), physical environments and administrative processes are essential to prevent dissatisfaction but do not inherently enhance satisfaction levels once a professional threshold is met (12, 18). In addition, although all inter-construct correlations remained below the commonly accepted threshold of 0.85, the moderate-to-strong correlations among the latent constructs indicate substantial overlap in explained variance. This overlap may partly explain the reduced unique contributions of some predictors within the structural model. Therefore, the non-significant effects observed for Procedures and Facilities should not be interpreted as evidence that these dimensions are unimportant, but rather that part of their explanatory power may be shared with other correlated constructs, particularly Personnel and Accessibility. Taken together, these findings highlight the multidimensional and interrelated nature of outpatient satisfaction, in which different service domains contribute jointly rather than independently to patients' overall perceptions.

Beyond these structural factors, patient perceptions were also significantly shaped by socio-demographic variations. Health Insurance (HI) expansion is a national policy cornerstone; our finding that HI holders report significantly higher satisfaction (*p* < 0.001) suggests that financial security enhances care perception. This is consistent with evidence from Indonesia, where HI facilitates service access and provides financial protection, directly fostering patient loyalty (19). This is particularly evident among lower-income groups, where HI acts as a critical equalizer by mitigating out-of-pocket expenses and amplifying the perceived value of care for vulnerable populations (13, 20, 21).

Geographical and experiential factors further refined these satisfaction patterns**.** A significant geographical gap was observed, with rural patients reporting higher satisfaction than those in remote regions (*p* < 0.001), likely due to differing expectations and perceived accessibility (22). Conversely, lower satisfaction in remote areas may reflect persistent systemic barriers in healthcare delivery despite national improvement efforts. Additionally, the significantly higher satisfaction among repeat visitors (*p* < 0.001) confirms that trust is a cumulative process. In the Vietnamese context, familiarity with hospital procedures and established provider-patient relationships significantly reduce anxiety, thereby enhancing overall satisfaction (12, 20). Furthermore, the substantial variation in satisfaction across different healthcare facilities (*p* < 0.001) reflects the diversity of management models. Public health centers generally reported higher mean scores than private facilities, potentially due to the advantages of low-cost access through health insurance and geographical proximity, which are high priorities for patients in developing regions. Conversely, lower scores in some private settings may stem from higher patient expectations relative to service costs, creating a wider gap between expectations and actual experience.

### 4.2 Bridging the gaps between expectations and experience: strategic improvements

The recommendation level consistently held the highest position among the three indices, while the total score (actual experience) ranked at the bottom. This underscores a disparity between high reputation-driven expectations and actual service delivery. This satisfaction-loyalty paradox, where patients are satisfied enough to recommend the facility despite minor dissatisfactions with procedures, is a phenomenon observed in both Chinese and Vietnamese healthcare studies, reflecting profound institutional trust despite infrastructure limitations (21). This trust is further evidenced by the temporal stability of recommendation levels shown in Figure 3. While overall satisfaction scores exhibited minor quarterly fluctuations—likely due to seasonal workload shifts—the intent to recommend remained high and unwavering. This suggests that patients' long-term loyalty is anchored in the hospital's established reputation, which effectively buffers against transient variations in daily service delivery.

Given that this resilient reputation is primarily built upon the quality of provider-patient interactions, prioritizing the enhancement of Personnel factor appears to be the most effective strategy to bridge the remaining experience gap. There is a need to enhance the soft skills of administrative and security staff, who often score lower than doctors (21, 23). Furthermore, Accessibility (*β* = 0.3, *p* < 0.001) requires investment in digital registration systems to reduce physical congestion, which our interaction analysis suggests would positively influence the patient's psychological receptivity to clinical care (24–27). Research indicates that reducing waiting times through improved process flow is a universal driver of outpatient satisfaction in both developed and developing regions (18).

### 4.3 Research contributions and innovations in the national assessement tool

This study provides a holistic view of the Vietnamese healthcare landscape by employing a multi-center approach across five diverse facilities (*N* = 2,500). Beyond its local impact, this research offers valuable lessons for other low- and middle-income countries (LMICs) striving to standardize patient feedback mechanisms. The successful implementation of a unified quality-monitoring framework in resource-constrained environments underscores the feasibility of institutionalizing patient-centered metrics as a formal performance indicator, moving beyond purely clinical outcomes (28).

A significant contribution of this work lies in the utilization and validation the updated version of the nationally standardized questionnaire, which introduced several strategic enhancements to improve measurement precision. Notably, making the “Phone number” field optional protects patient privacy and reduces social desirability bias, fostering more honest feedback (29). Furthermore, the inclusion of granular variables, such as “Place of residence,” “Family economic status,” and “Number of prior visits”, allows for a multi-dimensional analysis that was pivotal in identifying satisfaction gaps across different patient segments (30, 31).

Technically, the introduction of a “level 0” (non-applicable/no opinion) represents a major advancement in ensuring that mean values accurately reflect the experiences of direct service users without artificial inflation or deflation. Additionally, the integration of item E5 (value for money) and open-ended questions (G3, G4) provides both quantitative precision and qualitative depth. We recommend that hospital leaders prioritize the thematic analysis of these responses to identify specific pain points that structured scales might overlook, transforming the questionnaire into a practical tool for sustainable quality management (18, 32).

### 4.4 Limitations

Despite its contributions, this study faces several limitations. First, the cross-sectional design precludes causal inferences regarding satisfaction changes over time (33). Second, while the dataset is complete, the absence of missing data might reflect a “compliance tendency” among Vietnamese respondents, who may feel a sense of obligation to fill all items, potentially masking neutral or indifferent opinions (34, 35). Third, significant challenges were observed regarding the open-ended questions, specifically “requesting detailed reasons for any dissatisfaction” and “inviting suggestions to improve the hospital and the overall healthcare system”. Most respondents either left these sections blank or provided brief, non-substantive answers such as “None” or “No comment.” This lack of qualitative input could be attributed to the “survey burden” caused by the extensive length of the updated questionnaire, comprising 31 quantitative items plus additional demographics, which often leads to respondent fatigue (36, 37). Furthermore, the psychological urge to complete procedures quickly—such as obtaining medication or finalizing discharge—likely prompted patients to provide “pro forma” answers to save time (26). These factors created substantial difficulties in qualitative analysis, as the data sometimes failed to capture the true depth of patient aspirations or specific pain points. In addition, the socio-demographic characteristics of the study population should be interpreted in the context of the participating healthcare facilities rather than the general Vietnamese population. The observed socioeconomic profile and age distribution reflect the characteristics of patients attending the five participating facilities, which included both public and private healthcare providers across different regions of Vietnam. Therefore, these characteristics should not be interpreted as estimates of the national socioeconomic profile or age-specific disease burden. Lastly, although the study covered five facilities, the results may not be fully representative of the entire Vietnamese healthcare system. Future research should employ longitudinal designs and include a broader range of facility types to enhance generalizability (38).

## 5 Conclusion

This study confirms that the standardized questionnaire is a valid tool for assessing outpatient satisfaction in Vietnam. Based on data from 2,500 respondents, Personnel, Accessibility, and Outcomes were identified as the main associated factors, with staff attitude and communication showing the strongest influence. Although satisfaction scores were high, qualitative feedback remained limited. These findings support the need for policies that strengthen patient-centered care, improve service accessibility, and promote health insurance coverage as part of national healthcare quality improvement.

## Data Availability

The datasets presented in this article are not readily available because The data analyzed in this study is subject to the following licenses/restrictions: the raw data supporting the conclusions of this article are available from the participating healthcare facilities, including Minh Anh International Hospital, Saigon ITO Phu Nhuan Hospital, Tuy Phong Regional Health Center, Vinh Cuu Regional Health Center, and Nhan Hau Phu Cuong Clinic. However, access to these datasets is restricted due to institutional privacy policies and licensing agreements for the current study; therefore, they are not publicly available. Interested parties may obtain the data from the authors upon reasonable request, subject to formal permission from the respective healthcare facilities. Requests to access the datasets should be directed to haiyen@ump.edu.vn
