# Supplementary material for: Outpatient satisfaction assessed using a nationally standardized questionnaire: a survey of five health care facilities in Vietnam
Source: Front Health Serv. 2026 Aug 6;6:1844842. doi: 10.3389/frhs.2026.1844842 (PMC13490074; doi:10.3389/frhs.2026.1844842)
Supplement: Supplementary file 1 [file Datasheet1.pdf]

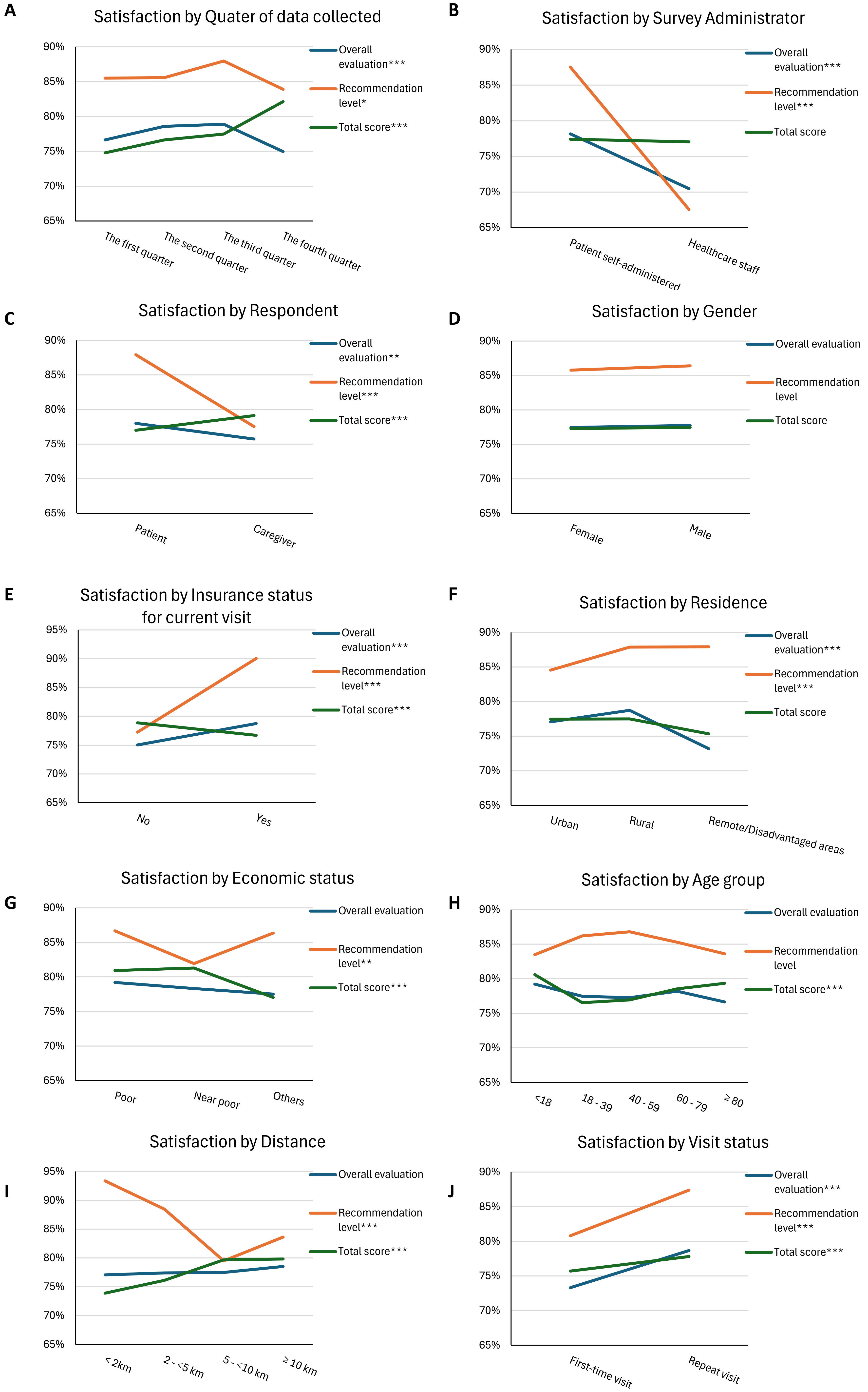

(*): $p < 0.05$, (**): $p < 0.01$, (***): $p < 0.001$

Suplementary Figure S1. Corresponding percentage of the means of general satisfaction indicators.

(A) Quarter of data collected; (B) Survey administrator; (C) Respondent; (D) Gender ; (E) Insurance status for current visit; (F) Residence; (G) Economic status; (H) Age; (I) Distance; (J) Visit status.
