# Supplementary material for: Outpatient satisfaction assessed using a nationally standardized questionnaire: a survey of five health care facilities in Vietnam
Source: Front Health Serv. 2026 Aug 6;6:1844842. doi: 10.3389/frhs.2026.1844842 (PMC13490074; doi:10.3389/frhs.2026.1844842)
Supplement: Supplementary file 3 [file Table4.docx]

| **Supplementary Table S1.** Sample size determination and participant recruitment at each participating healthcare facility | | | | | |
| --- | --- | --- | --- | --- | --- |
| **Healthcare facility** | **Average outpatient visits/day (N)** | **Finite population corrected**  **sample size (n_adj_)** | **Recommended sample size according to the Vietnam Ministry of Health guideline** | **Actual recruited participants (n)** | **Recruitment period** |
| Minh Anh International Hospital | 39 | 33 | 43 | 500 | Oct 2024–Sep 2025 |
| Saigon ITO Phu Nhuan Hospital | 120 | 75 | 44-62 | 500 | Oct 2024–Sep 2025 |
| Nhan Hau Phu Cuong Clinic | 95 | 65 | 44-62 | 500 | Oct 2024–Sep 2025 |
| Tuy Phong Regional Health Center | 240 | 109 | 74-109 | 500 | Oct 2024–June 2025 |
| Vinh Cuu Regional Health Center | 256 | 112 | 74-109 | 500 | Oct 2024–June 2025 |
| **Total** |  |  |  | **2500** |  |
| Note: *According to the Vietnam Ministry of Health guideline (Decision No. 56/QD-BYT, 2024), the minimum required sample size for each participating healthcare facility was determined through a three-step procedure: (1) calculation of the initial sample size (n = 196) using the single-proportion formula; (2) finite population correction based on the average number of outpatient visits per day (N); and (3) conversion of the corrected sample size to the corresponding standardized sample-size category specified in the guideline.* | | | | | |
