# Supplementary material for: Outpatient satisfaction assessed using a nationally standardized questionnaire: a survey of five health care facilities in Vietnam
Source: Front Health Serv. 2026 Aug 6;6:1844842. doi: 10.3389/frhs.2026.1844842 (PMC13490074; doi:10.3389/frhs.2026.1844842)
Supplement: Supplementary file 5 [file Table6.docx]

## **Supplement Table S3.** Statistical description of satisfaction items

| **Factors and items** | **Count** | **Percentage (%)** | **Valid percentage** | **M (SD)^a^** |
| --- | --- | --- | --- | --- |
| *Accessibility (Nhóm A)* |  |  |  |  |
| A1."directional signs" |  |  |  |  |
| Very dissatisfied = 1 | 4 | 0.16 | 0.16 | 3.98 (0.75) |
| Dissatisfied = 2 | 49 | 1.96 | 1.96 |  |
| Neutral = 3 | 554 | 22.16 | 22.16 |  |
| Satisfied = 4 | 1,286 | 51.44 | 51.44 |  |
| Very satisfied = 5 | 607 | 24.28 | 24.28 |  |
| I don't know = 0 | - | - | - |  |
| A2." clarity of department signs" |  |  |  |  |
| Very dissatisfied = 1 | 7 | 0.28 | 0.28 | 3.96 (0.77) |
| Dissatisfied = 2 | 38 | 1.52 | 1.52 |  |
| Neutral = 3 | 627 | 25.08 | 25.08 |  |
| Satisfied = 4 | 1,193 | 47.72 | 47.72 |  |
| Very satisfied = 5 | 635 | 25.40 | 25.40 |  |
| I don't know = 0 | - | - | - |  |
| A3."building layout" |  |  |  |  |
| Very dissatisfied = 1 | 4 | 0.16 | 0.16 | 4.01 (0.76) |
| Dissatisfied = 2 | 45 | 1.80 | 1.80 |  |
| Neutral = 3 | 551 | 22.04 | 22.04 |  |
| Satisfied = 4 | 1,222 | 48.88 | 48.88 |  |
| Very satisfied = 5 | 678 | 27.12 | 27.12 |  |
| I don't know = 0 | - | - | - |  |
| A4."corridor accessibility" |  |  |  |  |
| Very dissatisfied = 1 | 10 | 0.40 | 0.40 | 4.02 (0.78) |
| Dissatisfied = 2 | 42 | 1.68 | 1.68 |  |
| Neutral = 3 | 549 | 21.96 | 21.96 |  |
| Satisfied = 4 | 1,182 | 47.28 | 47.28 |  |
| Very satisfied = 5 | 717 | 28.68 | 28.68 |  |
| I don't know = 0 | - | - | - |  |
| A5."info convenience" |  |  |  |  |
| Very dissatisfied = 1 | 8 | 0.32 | 0.32 | 3.94 (0.83) |
| Dissatisfied = 2 | 102 | 4.08 | 4.08 |  |
| Neutral = 3 | 592 | 23.68 | 23.68 |  |
| Satisfied = 4 | 1,127 | 45.08 | 45.08 |  |
| Very satisfied = 5 | 671 | 26.84 | 26.84 |  |
| I don't know = 0 | - | - | - |  |
| *Procedures (Nhóm B)* |  |  |  |  |
| B1."process transparency" |  |  |  |  |
| Very dissatisfied = 1 | 11 | 0.44 | 0.44 | 3.82 (0.80) |
| Dissatisfied = 2 | 94 | 3.76 | 3.76 |  |
| Neutral = 3 | 712 | 28.48 | 28.48 |  |
| Satisfied = 4 | 1,197 | 47.88 | 47.88 |  |
| Very satisfied = 5 | 486 | 19.44 | 19.44 |  |
| I don't know = 0 | - | - | - |  |
| B2."Procedure convenience" |  |  |  |  |
| Very dissatisfied = 1 | 2 | 0.08 | 0.08 | 3.87 (0.77) |
| Dissatisfied = 2 | 84 | 3.36 | 3.36 |  |
| Neutral = 3 | 657 | 26.28 | 26.28 |  |
| Satisfied = 4 | 1,258 | 50.32 | 50.32 |  |
| Very satisfied = 5 | 499 | 19.96 | 19.96 |  |
| I don't know = 0 | - | - | - |  |
| B3."Pricing transparency" |  |  |  |  |
| Very dissatisfied = 1 | 5 | 0.20 | 0.20 | 3.86 (0.81) |
| Dissatisfied = 2 | 95 | 3.80 | 3.80 |  |
| Neutral = 3 | 703 | 28.12 | 28.12 |  |
| Satisfied = 4 | 1,135 | 45.40 | 45.40 |  |
| Very satisfied = 5 | 562 | 22.48 | 22.48 |  |
| I don't know = 0 | - | - | - |  |
| B4."Staff helpfulness" |  |  |  |  |
| Very dissatisfied = 1 | 29 | 1.16 | 1.16 | 3.65 (0.92) |
| Dissatisfied = 2 | 224 | 8.96 | 8.96 |  |
| Neutral = 3 | 811 | 32.44 | 32.44 |  |
| Satisfied = 4 | 966 | 38.64 | 38.64 |  |
| Very satisfied = 5 | 470 | 18.80 | 18.80 |  |
| I don't know = 0 | - | - | - |  |
| B5."staff attentiveness" |  |  |  |  |
| Very dissatisfied = 1 | 2 | 0.08 | 0.08 | 3.88 (0.80) |
| Dissatisfied = 2 | 73 | 2.92 | 2.92 |  |
| Neutral = 3 | 743 | 29.72 | 29.72 |  |
| Satisfied = 4 | 1,095 | 43.80 | 43.80 |  |
| Very satisfied = 5 | 587 | 23.48 | 23.48 |  |
| I don't know = 0 | - | - | - |  |
| B6."registration wait time" |  |  |  |  |
| Very dissatisfied = 1 | 3 | 0.12 | 0.12 | 3.88 (0.78) |
| Dissatisfied = 2 | 81 | 3.24 | 3.24 |  |
| Neutral = 3 | 671 | 26.84 | 26.84 |  |
| Satisfied = 4 | 1,200 | 48.00 | 48.00 |  |
| Very satisfied = 5 | 545 | 21.80 | 21.80 |  |
| I don't know = 0 | - | - | - |  |
| B7."waiting time for doctor" |  |  |  |  |
| Very dissatisfied = 1 | 14 | 0.56 | 0.56 | 3.88 (0.84) |
| Dissatisfied = 2 | 109 | 4.36 | 4.36 |  |
| Neutral = 3 | 627 | 25.08 | 25.08 |  |
| Satisfied = 4 | 1,157 | 46.28 | 46.28 |  |
| Very satisfied = 5 | 593 | 23.72 | 23.72 |  |
| I don't know = 0 | - | - | - |  |
| B8."consultation duration" |  |  |  |  |
| Very dissatisfied = 1 | 4 | 0.16 | 0.16 | 3.92 (0.81) |
| Dissatisfied = 2 | 101 | 4.04 | 4.04 |  |
| Neutral = 3 | 615 | 24.60 | 24.60 |  |
| Satisfied = 4 | 1,159 | 46.36 | 46.36 |  |
| Very satisfied = 5 | 621 | 24.84 | 24.84 |  |
| I don't know = 0 | - | - | - |  |
| B9."testing wait time" |  |  |  |  |
| Very dissatisfied = 1 | 6 | 0.24 | 0.24 | 3.86 (0.82) |
| Dissatisfied = 2 | 83 | 3.32 | 3.32 |  |
| Neutral = 3 | 739 | 29.56 | 29.56 |  |
| Satisfied = 4 | 1,087 | 43.48 | 43.48 |  |
| Very satisfied = 5 | 585 | 23.40 | 23.40 |  |
| I don't know = 0 | - | - | - |  |
| B10."result wait time" |  |  |  |  |
| Very dissatisfied = 1 | 6 | 0.24 | 0.24 | 3.87 (0.80) |
| Dissatisfied = 2 | 70 | 2.80 | 2.80 |  |
| Neutral = 3 | 746 | 29.84 | 29.84 |  |
| Satisfied = 4 | 1,107 | 44.28 | 44.28 |  |
| Very satisfied = 5 | 571 | 22.84 | 22.84 |  |
| I don't know = 0 | - | - | - |  |
| *Facilities (Nhóm C)* |  |  |  |  |
| C1."comfortable waiting area" |  |  |  |  |
| Very dissatisfied = 1 | 8 | 0.32 | 0.32 | 3.89 (0.77) |
| Dissatisfied = 2 | 73 | 2.92 | 2.92 |  |
| Neutral = 3 | 636 | 25.44 | 25.44 |  |
| Satisfied = 4 | 1,255 | 50.20 | 50.20 |  |
| Very satisfied = 5 | 528 | 21.12 | 21.12 |  |
| I don't know = 0 | - | - | - |  |
| C2."sufficient seating" |  |  |  |  |
| Very dissatisfied = 1 | 9 | 0.36 | 0.36 | 3.72 (0.78) |
| Dissatisfied = 2 | 84 | 3.36 | 3.36 |  |
| Neutral = 3 | 901 | 36.04 | 36.04 |  |
| Satisfied = 4 | 1,118 | 44.72 | 44.72 |  |
| Very satisfied = 5 | 388 | 15.52 | 15.52 |  |
| I don't know = 0 | - | - | - |  |
| C3."waiting room amenities" |  |  |  |  |
| Very dissatisfied = 1 | 5 | 0.20 | 0.20 | 3.81 (0.84) |
| Dissatisfied = 2 | 130 | 5.20 | 5.20 |  |
| Neutral = 3 | 735 | 29.40 | 29.40 |  |
| Satisfied = 4 | 1,096 | 43.84 | 43.84 |  |
| Very satisfied = 5 | 534 | 21.36 | 21.36 |  |
| I don't know = 0 | - | - | - |  |
| C4."entertainment facilities" |  |  |  |  |
| Very dissatisfied = 1 | 8 | 0.32 | 0.32 | 3.80 (0.85) |
| Dissatisfied = 2 | 118 | 4.72 | 4.72 |  |
| Neutral = 3 | 798 | 31.92 | 31.92 |  |
| Satisfied = 4 | 1,028 | 41.12 | 41.12 |  |
| Very satisfied = 5 | 548 | 21.92 | 21.92 |  |
| I don't know = 0 | - | - | - |  |
| C5."exam privacy" |  |  |  |  |
| Very dissatisfied = 1 | 5 | 0.20 | 0.20 | 3.80 (0.85) |
| Dissatisfied = 2 | 124 | 4.96 | 4.96 |  |
| Neutral = 3 | 787 | 31.48 | 31.48 |  |
| Satisfied = 4 | 1,023 | 40.92 | 40.92 |  |
| Very satisfied = 5 | 561 | 22.44 | 22.44 |  |
| I don't know = 0 | - | - | - |  |
| C6."clean restrooms" |  |  |  |  |
| Very dissatisfied = 1 | 25 | 1.00 | 1.00 | 3.62 (0.90) |
| Dissatisfied = 2 | 210 | 8.40 | 8.40 |  |
| Neutral = 3 | 888 | 35.52 | 35.52 |  |
| Satisfied = 4 | 940 | 37.60 | 37.60 |  |
| Very satisfied = 5 | 437 | 17.48 | 17.48 |  |
| I don't know = 0 | - | - | - |  |
| C7."green environment" |  |  |  |  |
| Very dissatisfied = 1 | 7 | 0.28 | 0.28 | 3.79 (0.83) |
| Dissatisfied = 2 | 129 | 5.16 | 5.16 |  |
| Neutral = 3 | 747 | 29.88 | 29.88 |  |
| Satisfied = 4 | 1,114 | 44.56 | 44.56 |  |
| Very satisfied = 5 | 503 | 20.12 | 20.12 |  |
| I don't know = 0 | - | - | - |  |
| C8."security & order" |  |  |  |  |
| Very dissatisfied = 1 | 2 | 0.08 | 0.08 | 3.85 (0.78) |
| Dissatisfied = 2 | 92 | 3.68 | 3.68 |  |
| Neutral = 3 | 681 | 27.24 | 27.24 |  |
| Satisfied = 4 | 1,228 | 49.12 | 49.12 |  |
| Very satisfied = 5 | 497 | 19.88 | 19.88 |  |
| I don't know = 0 | - | - | - |  |
| *Personnel* | | |  |  |
| D1."doctor communication" | | |  |  |
| Very dissatisfied = 1 | 12 | 0.48 | 0.48 | 3.83 (0.80) |
| Dissatisfied = 2 | 100 | 4.00 | 4.00 |  |
| Neutral = 3 | 687 | 27.48 | 27.48 |  |
| Satisfied = 4 | 1,214 | 48.56 | 48.56 |  |
| Very satisfied = 5 | 487 | 19.48 | 19.48 |  |
| I don't know = 0 | - | - | - |  |
| D2."support staff attitude" | | |  |  |
| Very dissatisfied = 1 | 13 | 0.52 | 0.52 | 3.75 (0.85) |
| Dissatisfied = 2 | 104 | 4.16 | 4.16 |  |
| Neutral = 3 | 897 | 35.88 | 35.88 |  |
| Satisfied = 4 | 965 | 38.60 | 38.60 |  |
| Very satisfied = 5 | 521 | 20.84 | 20.84 |  |
| I don't know = 0 | - | - | - |  |
| D3."fair treatment" | | |  |  |
| Very dissatisfied = 1 | 17 | 0.68 | 0.68 | 3.84 (0.86) |
| Dissatisfied = 2 | 115 | 4.60 | 4.60 |  |
| Neutral = 3 | 696 | 27.84 | 27.84 |  |
| Satisfied = 4 | 1,085 | 43.40 | 43.40 |  |
| Very satisfied = 5 | 587 | 23.48 | 23.48 |  |
| I don't know = 0 | - | - | - |  |
| D4."staff competence" | | |  |  |
| Very dissatisfied = 1 | 6 | 0.24 | 0.24 | 3.89 (0.85) |
| Dissatisfied = 2 | 118 | 4.72 | 4.72 |  |
| Neutral = 3 | 657 | 26.28 | 26.28 |  |
| Satisfied = 4 | 1,071 | 42.84 | 42.84 |  |
| Very satisfied = 5 | 648 | 25.92 | 25.92 |  |
| I don't know = 0 | - | - | - |  |
| *Outcomes* | | |  |  |
| E1."met expectations" | | |  |  |
| Very dissatisfied = 1 | 6 | 0.24 | 0.24 | 3.96 (0.77) |
| Dissatisfied = 2 | 61 | 2.44 | 2.44 |  |
| Neutral = 3 | 574 | 22.96 | 22.96 |  |
| Satisfied = 4 | 1,253 | 50.12 | 50.12 |  |
| Very satisfied = 5 | 606 | 24.24 | 24.24 |  |
| I don't know = 0 | - | - | - |  |
| E2."clear billing" | | |  |  |
| Very dissatisfied = 1 | 4 | 0.16 | 0.16 | 3.93 (0.80) |
| Dissatisfied = 2 | 63 | 2.52 | 2.52 |  |
| Neutral = 3 | 677 | 27.08 | 27.08 |  |
| Satisfied = 4 | 1,124 | 44.96 | 44.96 |  |
| Very satisfied = 5 | 632 | 25.28 | 25.28 |  |
| I don't know = 0 | - | - | - |  |
| E3."trust in quality" | | |  |  |
| Very dissatisfied = 1 | 1 | 0.04 | 0.04 | 4.01 (0.75) |
| Dissatisfied = 2 | 52 | 2.08 | 2.08 |  |
| Neutral = 3 | 536 | 21.44 | 21.44 |  |
| Satisfied = 4 | 1,254 | 50.16 | 50.16 |  |
| Very satisfied = 5 | 657 | 26.28 | 26.28 |  |
| I don't know = 0 | - | - | - |  |
| E4."price satisfaction" | | |  |  |
| Very dissatisfied = 1 | 2 | 0.08 | 0.08 | 4.05 (0.77) |
| Dissatisfied = 2 | 52 | 2.08 | 2.08 |  |
| Neutral = 3 | 515 | 20.60 | 20.60 |  |
| Satisfied = 4 | 1,171 | 46.84 | 46.84 |  |
| Very satisfied = 5 | 760 | 30.40 | 30.40 |  |
| I don't know = 0 | - | - | - |  |
| *General satisfaction indicators* |  |  |  |  |
| G1.“patient’s overall evaluation of this hospital” (overall evaluation) | | |  |  |
| Satisfaction score from 0 to 20% = 1 | 8 | 0.32 | 0.32 | 77.59% (15.34%) |
| Satisfaction score from 21 to 40% = 2 | 56 | 2.24 | 2.24 |  |
| Satisfaction score from 41 to 60% = 3 | 369 | 14.76 | 14.76 |  |
| Satisfaction score from 61 to 80% = 4 | 984 | 39.36 | 39.36 |  |
| Satisfaction score from 81 to 100% = 5 | 1,083 | 43.32 | 43.32 |  |
| G2.“Return or recommendation of this hospital to others” (recommendation level) | | |  |  |
| Certainly never return = 1 | 28 | 1.12 | 1.12 | 4.30 (0.85) |
| Return due to limited alternatives = 2 | 126 | 5.04 | 5.04 |  |
| Intent to switch to other facilities =3 | 89 | 3.56 | 3.56 |  |
| Probably will return = 4 | 1,074 | 42.96 | 42.96 |  |
| Certainly will return or recommend to others = 5 | 1,183 | 47.32 | 47.32 |  |
| Others = 6 | - | - | - |  |
| The M (SD) calculation excluded all options with a "0" value | | | |  |
