# Supplementary material for: Outpatient satisfaction assessed using a nationally standardized questionnaire: a survey of five health care facilities in Vietnam
Source: Front Health Serv. 2026 Aug 6;6:1844842. doi: 10.3389/frhs.2026.1844842 (PMC13490074; doi:10.3389/frhs.2026.1844842)
Supplement: Supplementary file 6 [file Table7.docx]

**Supplementary Table S4.** STROBE Statement – checklist of items included in reports of observational study

|  | Item No. | Recommendation | Page  No. | Relevant text from manuscript | |
| --- | --- | --- | --- | --- | --- |
| **Title and abstract** | 1 | (*a*) Indicate the study’s design with a commonly used term in the title or the abstract | 1 | Outpatient satisfaction assessed using a nationally standardized questionnaire:  a survey of five health care facilities in Vietnam | |
|  |  | (*b*) Provide in the abstract an informative and balanced summary of what was done and what was found | 1 | Background, Method, Results,Conclusion | |
| Introduction | | | | |  |
| Background/rationale | 2 | Explain the scientific background and rationale for the investigation being reported | 2 | 1 Introduction  Patient satisfaction is a key indicator of healthcare quality and system performance  ... Recently, the framework was updated via Decision No. 56/QD-BYT on January 8, 2024  ... This mandate introduced standardized survey templates for assessing the satisfaction of both patients and healthcare providers, officially superseding models from Decision No. 4448/QD-BYT (2013) and Decision No. 3869/QD-BYT (2019) ... There is a notable lack of evidence-based research evaluating diverse healthcare models simultaneously - from private international hospitals in urban centers to public district medical centers and provincial clinics ... Furthermore, few studies have employed robust statistical methods like Structural Equation Modeling (SEM) to validate this 2024 version ... This study aims to address these gaps by analyzing a sample of 2,500 outpatients to to identify key satisfaction determinants | |
| Objectives | 3 | State specific objectives, including any prespecified hypotheses | 2 | 1. Introduction  This study aims to address these gaps by analyzing a sample of 2,500 outpatients to to identify key satisfaction determinants. Using a nationally standardized questionnaire, the research examines how demographic and service-related factors contribute to the perceived quality of care, offering actionable recommendations for healthcare improvement in Vietnam | |
| Methods | | | | |  |
| Study design | 4 | Present key elements of study design early in the paper | 3 | 2.1 Study design and setting | |
| Setting | 5 | Describe the setting, locations, and relevant dates, including periods of recruitment, exposure, follow-up, and data collection | 3 | 2.1 Study design and setting  2.2 Participants and study procedures | |
| Participants | 6 | (*a*) *Cohort study*—Give the eligibility criteria, and the sources and methods of selection of participants. Describe methods of follow-up  *Case-control study*—Give the eligibility criteria, and the sources and methods of case ascertainment and control selection. Give the rationale for the choice of cases and controls  *Cross-sectional study*—Give the eligibility criteria, and the sources and methods of selection of participants | 3 | 2.2 Participants and study procedures  Data collection occurred between October 2024 and September 2025.  Eligible participants included all outpatients who had completed the clinical examination process and were either waiting for payment or medication.  Convenience sampling was applied to recruit participants. Following an explanation of the study’s objectives, participants provided written informed consent before completing the survey via a paper-based questionnaire or a digital interface (tablet/smartphone) accessed via a QR code. | |
|  |  | (*b*) *Cohort study*—For matched studies, give matching criteria and number of exposed and unexposed  *Case-control study*—For matched studies, give matching criteria and the number of controls per case |  | Not applicable. | |
| Variables | 7 | Clearly define all outcomes, exposures, predictors, potential confounders, and effect modifiers. Give diagnostic criteria, if applicable | 4 | 2.4 Measuring tool | |
| Data sources/ measurement | 8* | For each variable of interest, give sources of data and details of methods of assessment (measurement). Describe comparability of assessment methods if there is more than one group | *4-5* | 2.5 Statistical Analysis | |
| Bias | 9 | Describe any efforts to address potential sources of bias | 3, 5 | 2.2. Participants and study procedures  Participation was entirely voluntary, and anonymity was strictly maintained to minimize social desirability bias. (*Psychological Bias)*  2.5.2. Data analysis  Harman’s single-factor test was applied to assess common method variance. (*Common Method Bias)* | |
| Study size | 10 | Explain how the study size was arrived at | 3-4 | 2.3. Sample size determination  The sample size was determined following the national standards for healthcare quality appraisal as mandated in Decision No. 56/QD-BYT (2024)... The minimum required sample (n) was calculated using the formula for a single proportion: $n=Z_{(1-\frac{\alpha}{2})}^{2}.\frac{p(1-p)}{d^{2}}$ ... A total of 2,500 participants were recruited... significantly exceeding the minimum cumulative requirement. | |

Continued on next page

| Quantitative variables | 11 | Explain how quantitative variables were handled in the analyses. If applicable, describe which groupings were chosen and why | 4-5 | 2.5.2 Data analysis |
| --- | --- | --- | --- | --- |
| Statistical methods | 12 | (*a*) Describe all statistical methods, including those used to control for confounding | 4-5 | 2.5.2 Data analysis  Statistical analyses were performed using IBM SPSS 27.0 and AMOS 24.0... Descriptive statistics, including frequencies, percentages, means and standard deviations (SD), were used... Prior to group comparisons, normality tests were conducted... One-way ANOVA, Welch ANOVA, or Kruskal-Wallis test were utilized... A two-step Structural Equation Modeling (SEM) approach was adopted... |
|  |  | (*b*) Describe any methods used to examine subgroups and interactions | 4-5 | 2.5.2 Data analysis  ...one-way ANOVA was employed to identify significant differences in satisfaction scores across demographic variables such as age, gender, insurance status, and facility types. |
|  |  | (*c*) Explain how missing data were addressed | 4 | 2.5.1 Data preparation All 2,500 collected questionnaires were strictly screened for completeness; as all responses were fully completed without missing values, the entire sample (N=2,500) was retained for analysis. |
|  |  | (*d*) *Cohort study*—If applicable, explain how loss to follow-up was addressed  *Case-control study*—If applicable, explain how matching of cases and controls was addressed  *Cross-sectional study*—If applicable, describe analytical methods taking account of sampling strategy |  | Not applicable. |
|  |  | (*e*) Describe any sensitivity analyses |  | Not applicable. |
| Results | | | | |
| Participants | 13* | (a) Report numbers of individuals at each stage of study—eg numbers potentially eligible, examined for eligibility, confirmed eligible, included in the study, completing follow-up, and analysed | 4 | 2.5.1. Data preparation  A total of 2,500 outpatients participated in the study. All 2,500 collected questionnaires were strictly screened for completeness; as all responses were fully completed without missing values, the entire sample (N=2,500) was retained for analysis. |
|  |  | (b) Give reasons for non-participation at each stage | 3 | 2.2 Participants and study procedures  Participation was entirely voluntary. |
|  |  | (c) Consider use of a flow diagram |  | Not applicable. |
| Descriptive data | 14* | (a) Give characteristics of study participants (eg demographic, clinical, social) and information on exposures and potential confounders | 3-4 | 3.1 Socio-demographic characteristics of patients |
|  |  | (b) Indicate number of participants with missing data for each variable of interest | 6-7 | Table 1 (no missing data) |
|  |  | (c) *Cohort study*—Summarise follow-up time (eg, average and total amount) |  | Not applicable. |
| Outcome data | 15* | *Cohort study*—Report numbers of outcome events or summary measures over time |  | Not applicable. |
|  |  | *Case-control study—*Report numbers in each exposure category, or summary measures of exposure |  | Not applicable. |
|  |  | *Cross-sectional study—*Report numbers of outcome events or summary measures | *7* | 3.2 Structural equation modelling analysis, Figure 1, Table 2 |
| Main results | 16 | (*a*) Give unadjusted estimates and, if applicable, confounder-adjusted estimates and their precision (eg, 95% confidence interval). Make clear which confounders were adjusted for and why they were included | 7-9 | 3.2.1. Validity and reliability  3.2.2. Evaluation of measurement and structural models  3.3. Satisfaction level of outpatients  Figure 2 |
|  |  | (*b*) Report category boundaries when continuous variables were categorized |  | Not applicable. |
|  |  | (*c*) If relevant, consider translating estimates of relative risk into absolute risk for a meaningful time period |  | Not applicable. |

Continued on next page

| Other analyses | 17 | Report other analyses done—eg analyses of subgroups and interactions, and sensitivity analyses | 9-12 | 3.4. Association between socio-demographics factors and general satisfaction levels  3.5. Subgroup analysis of key satisfaction-related factors  Figure 3, Figure 4, Table 3 |
| --- | --- | --- | --- | --- |
| Discussion | | | | |
| Key results | 18 | Summarise key results with reference to study objectives | 13-16 | 4. Discussion  The study successfully identified the primary influencing factors: personnel, accessibility, and outcomes... Personnel, Accessibility, and Outcomes are the three primary pillars. |
| Limitations | 19 | Discuss limitations of the study, taking into account sources of potential bias or imprecision. Discuss both direction and magnitude of any potential bias | 16 | 4.3. Limitation  First, the cross-sectional design precludes causal inferences... Second, the absence of missing data might reflect a 'compliance tendency'... Third, survey burden... led to respondent fatigue in open-ended questions. |
| Interpretation | 20 | Give a cautious overall interpretation of results considering objectives, limitations, multiplicity of analyses, results from similar studies, and other relevant evidence | 13-14 | Specifically, the primary contribution of Personnel (β = 0.43, p < 0.001) echoes Nguyen et al. (2022) regarding staff expertise in Vietnam, while the association of Accessibility (β = 0.30, p < 0.001) parallels Hoang et al. (2023), highlighting administrative efficiency as a key differentiator in multi-center settings .... This satisfaction-loyalty paradox, where patients are satisfied enough to recommend the facility despite minor dissatisfactions with procedures, is a phenomenon observed in both Chinese and Vietnamese healthcare studies, reflecting profound institutional trust despite infrastructure limitations. |
| Generalisability | 21 | Discuss the generalisability (external validity) of the study results | 15 | This study provides a holistic view of the Vietnamese healthcare landscape by employing a multi-center approach across five diverse facilities (N = 2,500). Beyond its local impact, this research offers valuable lessons for other low- and middle-income countries (LMICs) striving to standardize patient feedback mechanisms. |
| Other information | |  | | |
| Funding | 22 | Give the source of funding and the role of the funders for the present study and, if applicable, for the original study on which the present article is based | 17 | Funding  The author(s) declare that no financial support was received for the research, authorship, and/or publication of this article. |

*Give information separately for cases and controls in case-control studies and, if applicable, for exposed and unexposed groups in cohort and cross-sectional studies.
