# Supplementary material for: Outpatient satisfaction assessed using a nationally standardized questionnaire: a survey of five health care facilities in Vietnam
Source: Front Health Serv. 2026 Aug 6;6:1844842. doi: 10.3389/frhs.2026.1844842 (PMC13490074; doi:10.3389/frhs.2026.1844842)
Supplement: Supplementary file 7 [file Table5.docx]

# **Supplementary material**

## **Supplement Table S2.** Outpatient Satisfaction Questionnaire
